# Functional Insight into and Refinement of the Genomic Boundaries of the *JARID2*-Neurodevelopmental Disorder Episignature

**DOI:** 10.3390/ijms241814240

**Published:** 2023-09-18

**Authors:** Liselot van der Laan, Kathleen Rooney, Sadegheh Haghshenas, Ananília Silva, Haley McConkey, Raissa Relator, Michael A. Levy, Irene Valenzuela, Laura Trujillano, Amaia Lasa-Aranzasti, Berta Campos, Neus Castells, Eline A. Verberne, Saskia Maas, Mariëlle Alders, Marcel M. A. M. Mannens, Mieke M. van Haelst, Bekim Sadikovic, Peter Henneman

**Affiliations:** 1 Department of Human Genetics, Amsterdam Reproduction and Development Research Institute, Amsterdam University Medical Center, University of Amsterdam, Meibergdreef 9, 1105 AZ Amsterdam, The Netherlands; 2 Verspeeten Clinical Genome Centre, London Health Sciences Centre, London, ON N6A 5W9, Canadaraissa.relator@lhsc.on.ca (R.R.);; 3 Department of Pathology and Laboratory Medicine, Western University, London, ON N6A 3K7, Canada; 4 Medicine Genetics Group, Vall d’Hebron Institut de Recerca (VHIR), Vall d’Hebron Barcelona Hospital Campus, Vall d’Hebron Hospital Universitari, 129, 08035 Barcelona, Spain; 5 Department of Clinical and Molecular Genetics, Vall d’Hebron Barcelona Hospital Campus, Vall d’Hebron Hospital Universitari, 129, 08035 Barcelona, Spain

**Keywords:** JARID2, CNV, DNA methylation, episignature, intellectual disability

## Abstract

*JARID2* (Jumonji, AT-rich interactive domain 2) haploinsufficiency is associated with a clinically distinct neurodevelopmental syndrome. It is characterized by intellectual disability, developmental delay, autistic features, behavior abnormalities, cognitive impairment, hypotonia, and dysmorphic features. *JARID2* acts as a transcriptional repressor protein that is involved in the regulation of histone methyltransferase complexes. *JARID2* plays a role in the epigenetic machinery, and the associated syndrome has an identified DNA methylation episignature derived from sequence variants and intragenic deletions involving *JARID2*. For this study, our aim was to determine whether patients with larger deletions spanning beyond *JARID2* present a similar DNA methylation episignature and to define the critical region involved in aberrant DNA methylation in 6p22–p24 microdeletions. We examined the DNA methylation profiles of peripheral blood from 56 control subjects, 13 patients with (likely) pathogenic *JARID2* variants or patients carrying copy number variants, and three patients with *JARID2* VUS variants. The analysis showed a distinct and strong differentiation between patients with (likely) pathogenic variants, both sequence and copy number, and controls. Using the identified episignature, we developed a binary model to classify patients with the *JARID2*-neurodevelopmental syndrome. DNA methylation analysis indicated that *JARID2* is the driver gene for aberrant DNA methylation observed in 6p22–p24 microdeletions. In addition, we performed analysis of functional correlation of the *JARID2* genome-wide methylation profile with the DNA methylation profiles of 56 additional neurodevelopmental disorders. To conclude, we refined the critical region for the presence of the *JARID2* episignature in 6p22–p24 microdeletions and provide insight into the functional changes in the epigenome observed when regulation by *JARID2* is lost.

## 1. Introduction

*JARID2* (OMIM; #601594) haploinsufficiency (DIDDF, OMIM; #620098) leads to a clinically distinct neurodevelopmental syndrome characterized by intellectual disability (ID), developmental delay (DD), autistic features, behavior abnormalities, cognitive impairment, hypotonia, and dysmorphic features such as high anterior hairline, deeply set eyes, depressed nasal bridge, full lips, broad forehead, and bulbous nasal tip [1,2]. *JARID2* is located at chromosome region 6p22.3. Large multi-gene deletions of chromosome region 6p22–p24 involving *JARID2* have been described in individuals who present with a similar phenotype as those with *JARID2* intragenic deletions and loss-of-function variants [3]. Baroy et al. assessed variable-sized deletions of this region and implicated the chromatin remodelers *JARID2* and *ATXN1* as likely disease-causing candidate genes [3]. 

*JARID2* functions as a transcriptional repressor protein involved in the regulation of various histone methyltransferase complexes. The JARID2 protein also plays a crucial role in the recruitment and activation of the polycomb repressive complex 2 (PRC2). PCR2 is a complex that suppresses the expression of target genes on histone H3 lysine 27 (H3K27) methylation [4,5]. 

Recently, our group has reported evidence of *JARID2* involvement in the epigenetic regulation of DNA methylation by demonstrating a highly sensitive and specific DNA methylation episignature in the peripheral blood of affected patients [6]. This episignature biomarker was trained using single-nucleotide variants (SNV) or intragenic deletions of *JARID2* and did not include larger multi-gene copy number variants (CNVs) that are part of the overlapping 6p22–p24 microdeletion syndrome. Disruption of multiple genes in this region may impact the phenotype and result in a different episignature from that observed in individuals with variants limited to *JARID2* [7]. Several episignatures have been defined for chromosomal microdeletion/duplication syndromes, where episignature profiles have been attributed to a specific gene locus [7,8]. Similarly, this approach can be applied to identify a target gene within a larger CNV responsible for DNA methylation changes, providing valuable insights into the pathophysiology of CNV disorders and identifying new candidate genes that are responsible for the phenotypic features [7]. For example, our group previously demonstrated that the *HNRNPU* episignature included two cases with a large CNV spanning regions involving distinct genes next to *HNRPNU* [8]. Here, it was shown that including individuals carrying distinct regional CNVs in episignature assessment and discovery is a powerful method for identifying the causal gene within the deletion region for a given disorder. 

In this study, we hypothesized that patients with 6p22–p24 microdeletion syndrome encompassing *JARID2* may exhibit DNA methylation episignatures overlapping with those seen in patients with SNVs and intragenic CNVs [7]. Our study aims to refine the critical region for the *JARID2* episignature, among other potential epigenetic regulatory genes within the 6p22–p24 region. Finally, we offer novel insights into the global genomic DNA methylation architecture of *JARID2* and compare *JARID2* to 56 other neurodevelopmental episignature disorders.

## 2. Results

### 2.1. JARID2 Molecular and Clinical Information 

**Figure 1:**
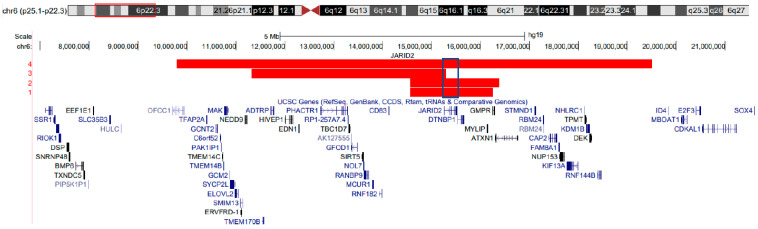
**Genomic region of the large multi-gene deletions involving *JARID2* in this cohort.** Patient 3 had a large deletion including multiple genes, however, including exon 1 of *JARID2*. Deletions in other cases encompass the entire JARID2 coding sequence. Cytogenetic bands and known genes are presented in this figure using the UCSC genome browser 2009 (GRCH37/hg19) genome build [9].

The molecular and clinical details of the CNV cohort are summarized in Table 1 and Figure 1. All four individuals have a large deletion that includes *JARID2* and at least two additional genes. Whereas cases 1–2 and 4 fully encompass *JARID2*, case 3 has a large multi-gene deletion that includes only exon 1 of *JARID2*.

Table 1 shows the clinical details of the CNV cohort, with a summary of the cases from the discovery of the *JARID2*-neurodevelopmental syndrome episignature [6]. The cases with CNV deletions had ID, DD, behavioral abnormalities, and autistic features. Furthermore, neurologic examination showed that two cases presented with hypotonia, and one had MRI abnormalities overlapping with the original discovery cohort. Dysmorphic features were present in all patients, and percent overlap with the discovery cohort was indicated.

### 2.2. Identification and Assessment of an Episignature for JARID2 

**Figure 2:**
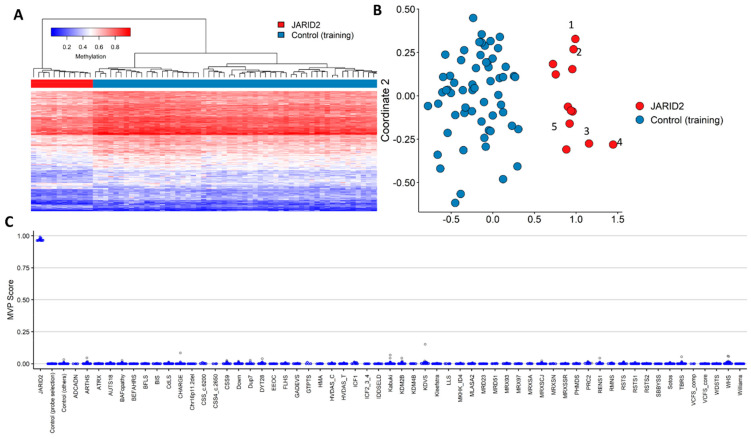
Hypomethylated episignature for *JARID2*-neurodevelopmental syndrome. Including five new and seven previously described cases. (**A**) Euclidean clustering heatmap of the cases in red and the controls in blue. Rows of the heatmap correspond to the selected probes for the identification of the episignature, and the columns represent the cases and controls. The methylation levels are colored to show the intensity values, with 0 as blue and 1 as red. (**B**) Two-dimensional multidimensional scaling plot of the patients in red and the controls in blue. The *x*- and *y*-axis represent the first and second dimension of the output (Coordinate 1 and 2, respectively). (**C**) The support vector machine classifier was trained using the discovered signature probes as features to predict class probability of the training cases. We trained the model using the initial training cohort and their controls. Seventy-five percent of the renaming EKD samples with other known disorders and matched episignature were used, as well as unaffected controls. The remaining 25% from the EKD were used as test samples. We performed these four times, so every sample was tested once, and we used the average MVP scores for each test (gray) and training (blue).

We assessed CNV cases and one additional SNV case using the previously derived *JARID* episignature. All cases were positive for a common *JARID2* episignature through MDS and heatmap clustering (Supplementary Figure S1). Subsequently, we combined all cases and performed an extended episignature discovery analysis. Here, we included the four CNV and one new SNV case and eight previously described patients with pathogenic sequence variants within *JARID2* [6]. All study samples passed quality control, and the feature selection procedure yielded 218 probes (Supplementary Table S2), which showed distinct clustering between cases and controls. Hierarchical clustering (heatmap) and MDS showed clear separation between this cohort and matched controls (Figure 2A,B). Using twelve rounds of leave-one-out cross-validation followed by unsupervised hierarchical and MDS clustering (Supplementary Figure S2), we demonstrated reproducibility for the combined *JARID2* episignature. Lastly, an SVM model was constructed that showed an MVP score close to 1 for all but one case, indicating high sensitivity and specificity of the model (Figure 2C). 

**Figure 3:**
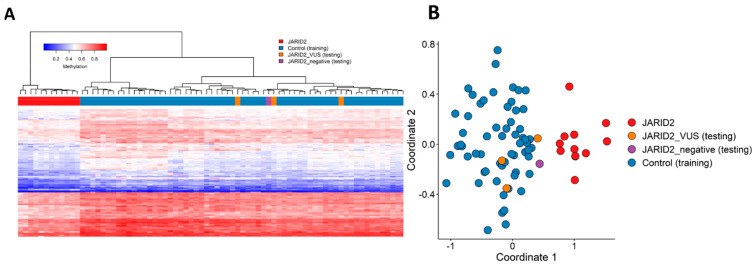
**Assessment of the VUSs and the *JARID2*_negative cases relative to twelve episignature positive cases.** (**A**) We created a Euclidean clustering heatmap of the cases in red, 3 VUS patients in orange, and the controls in blue. Rows of the heatmap correspond to the selected probes for the identification of the episignature, and the columns represent the cases and controls. The methylation levels are colored to show the intensity values, with 0 as blue and 1 as red. (**B**) Two-dimensional multidimensional scaling plot of the patients in red, 3 VUS patients in orange, JARID_negative cases in purple, and the controls in blue. The *x*- and *y*-axis represent the first and second dimension of the output (Coordinate 1 and 2, respectively). The 3 VUS patients in orange and the JARID2_negative case in purple were all clustered with the controls.

The one case (#8, from Verberne et al.) that did not map to the *JARID2* episignature was excluded from the episignature discovery cohort (JARID2_negative) (Figure 3). The three individuals carrying a *JARID2* missense variant of uncertain significance (VUS) were not included in episignature discovery and were assessed separately as testing samples by plotting them alongside the affected cases with CNVs and SNVs in *JARID2* and controls, using the same selected probes. The three individuals with a VUS and the JARID2_negative were clustered with controls, indicating the absence of the *JARID2* episignature (Figure 3). 

### 2.3. Annotation of the Global JARID2 DNA Methylation Profile and Correlation to the 56 Neurodevelopmental Disorder Episignatures on EpiSign™

**Figure 4:**
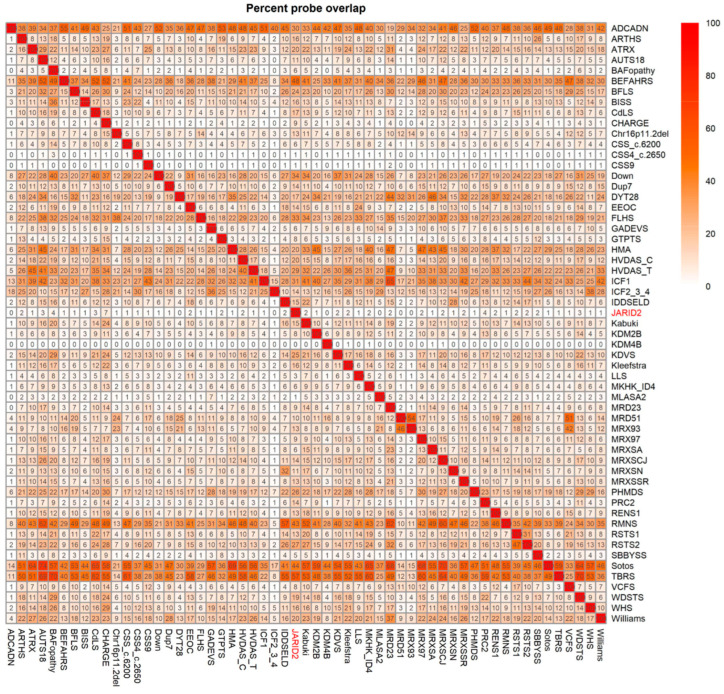
The DMPs shared between the *JARID2* (highlighted in red) cohort and the other 56 EpiSign^TM^ disorders with known episignatures. All DMPs were used in calculating the overlap percentage. Heatmap showing the percentage of overlap between probes for each cohort. Colors indicate the percentage of the *y*-axis cohort’s probes that are also found in the *x*-axis cohort’s probes.

We conducted a clustering analysis using the top number of differentially methylated probes (DMP) for all the cohorts described earlier by Levy et al. [10] to uncover relationships between those cohorts irrespective of the number of selected DMPs. We identified a genome-wide DMP set for *JARID2* based on differential DNA methylation and p-value relative to age-, sex-, and array-matched controls from the EpiSign Knowledge Database (EKD). We then compared this list to the genome-wide DMP list of the other 56 EpiSign^TM^ V3 classifier episignature disorders, as described before by Levy et al. [10] (Figure 4). The *JARID2* probe set comprised 628 DMPs, with the DMPs range for all cohorts spanning from 279 to 151848. Notably, *JARID2* exhibited the highest overlap with CHARGE (~7%, CHD7), BAFopathy (~4%, including *ARID1A*, *ARID1B*, *SMARCB1*, *SMARCA2*, *SMARCA4*), and the PCR2 complex, which houses Cohen–Gibson syndrome (COGIS) and Weaver syndrome (WVS) (~4%, *EED*, *EZH2)* (Figure 4). The circos plot visually represents a similar overlap represented in the heatmap, with the thickness of the lines indicating the number of DMPs shared between the two cohorts (Supplementary Figure S3). 

**Figure 5:**
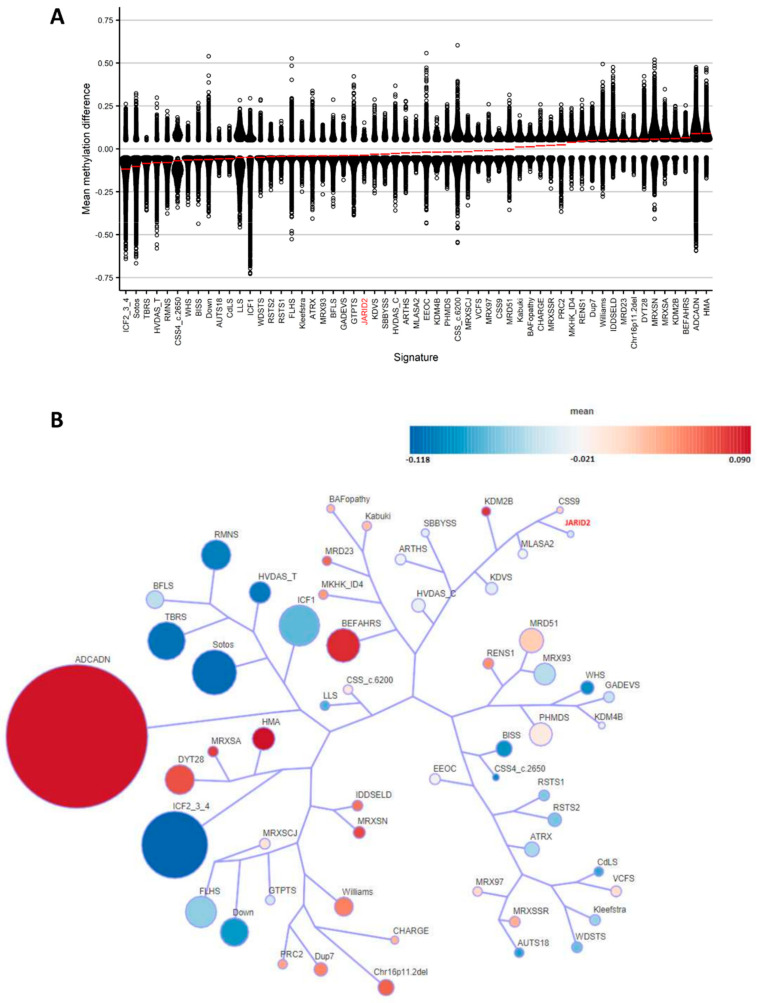
Relationship between the *JARID2* (highlighted in red) cohort and the 56 other neurodevelopmental conditions on the EpiSign^TM^ clinical classifier. Five hundred most significant DMPs for each signature. (**A**) Relative mean methylation differences of all DMPs for each cohort sorted by mean methylation. Circles represent unique probes. Red lines indicate mean methylation. (**B**) Tree-and-leaf visualization of Euclidean clustering of 56 cohorts using the top DMPs for each group. Cohort samples were aggregated using the median value of each probe within a group. A leaf node represents a cohort, with node sizes illustrating relative scales of the number of selected DMPs for the corresponding cohort, and node colors are indicative of the global mean methylation difference.

Next, we conducted a correlation analysis between the *JARID2* cohort and the 56 episignature conditions (Figure 5). We compared the mean DMP beta-values for each cohort, revealing that *JARID2* exhibited relative global hypomethylation (Figure 5A). To assess the similarity in genome-wide methylation profiles, we utilized the top 500 DMPs for each cohort. When cohorts had less than 500 DMPs, all DMPs were used in the analysis. A tree-and-leaf plot showed that the *JARID2* genome-wide DNA methylation change is most closely related to the DNA methylation changes of Coffin–Siris syndrome-9 (CSS9; *SOX11*), myopathy, lactic acidosis, and sideroblastic anemia 2 (MLASA2; *YARS2*) and Lysine-demethylase 2B (*KDM2B*) (Figure 5B).

### 2.4. Detection of Differentially Methylated Regions 

Detection of differentially methylated regions (DMRs) was based on non-trained differentially methylated positions using DMRcate. Here, we identified two significantly hypomethylated DMRs for the JARID2 cohort (Supplementary Table S3). Both of the DMRs were located within a CpG island that covered a promotor region. The first DMR was annotated to chromosome 1 and involved *HOXA-AS3*, *HOXA3*, *RP1-170O19.22*, *HOXA5*, and *HOXA6* gene clusters, and the second DMR was on chromosome 17 and overlapped with the *RP11-1055B8.6*, *RP11-1055B8.7*, and *MIR4740* genes.

### 2.5. Genomic Location of Classifying DMPs and DMRs 

**Figure 6:**
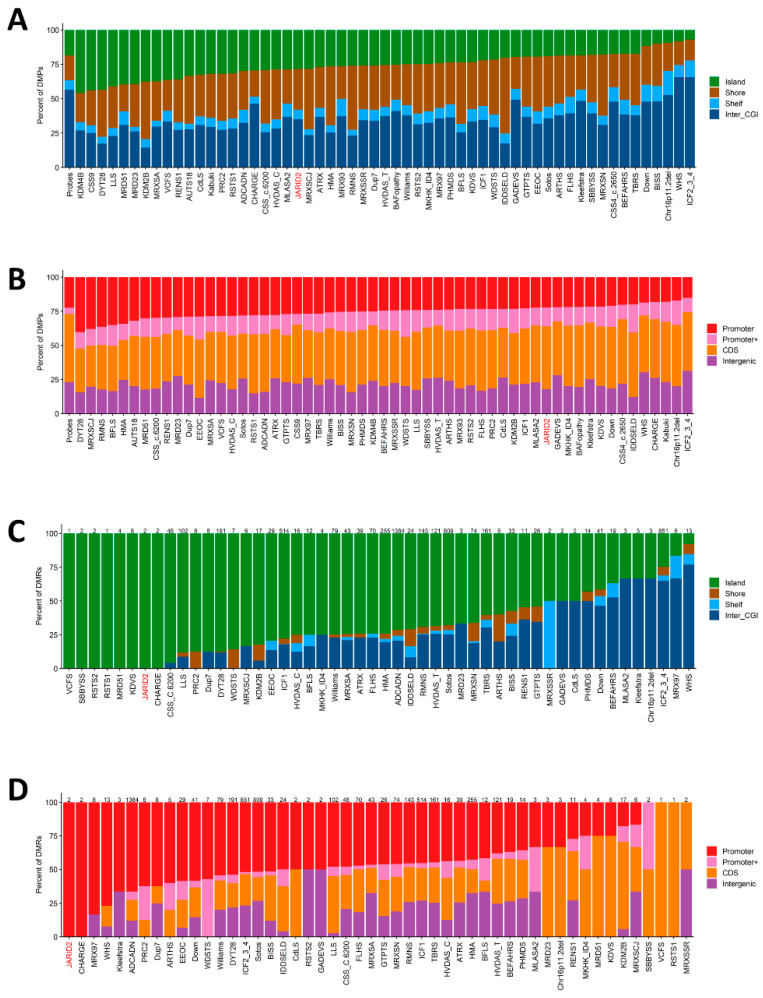
**The annotated DMPs and DMRs in the context of CpG islands and genes.** (**A**) DMPs annotated in the context of CpG islands and (**B**) DMPs annotated in the context of genes. (**C**) DMRs annotated in the context of CpG islands and (**D**) DMRs annotated in the context of genes.

We proceeded to investigate the genomic location of the DMPs and DMRs concerning CpG islands and genes. Figure 6A illustrates that DMPs are predominantly situated in genomic regions outside of the CpG islands and their shore/shelf regions. Similarly, concerning genes, we observed an enrichment of DMPs in coding regions and intergenic regions, with fewer occurrences in promoter regions (Figure 6B). In contrast, both DMRs were annotated to CpG islands (Figure 6C) and, in relation to genes, were located in the promotor regions of the *HOXA-AS3*, *HOXA3*, *RP1-170O19.22*, *HOXA5*, *HOXA6*, *RP11-1055B8.6*, *RP11-1055B8.7*, and *MIR4740* genes (Figure 6D). Furthermore, we noted a significant difference in the distribution of DMPs in the *JARID2* profile compared to the background probe distribution concerning genes (*p*-value < 7.06 × 10^−11^) and CpG islands (*p*-value < 2.98 × 10^−28^).

## 3. Discussion

DNA methylation episignatures can be utilized for the molecular diagnosis of individuals with Mendelian neurodevelopmental disorders and for the assessment of ambiguous genetic findings such as VUS reclassification. The list of episignature disorders is rapidly expanding, with over 70 episignatures having currently been reported [11]. 

The aim of this study was to investigate whether large CNVs containing *JARID2* exhibit the same DNA methylation pattern as those previously described for intragenic variants and to refine the critical region for the presence of the *JARID2* episignature in microdeletions involving 6p22–p24. Additionally, we aimed to further explore the overlap of the global methylation profile of affected *JARID2* cases with other Mendelian disorders with known episignatures. We have demonstrated that multi-gene CNVs including *JARID2* display the same DNA methylation episignature as intragenic variants in *JARID2*. Moreover, we have established the genomic 6p22–p24 deletion boundaries for an episignature that encompasses both sequence and copy number variants. Case 4 possesses the largest deletion and includes four other genes related to epigenetic regulation: *TFAP2A*, *SIRT5*, *ATXN1,* and *KDM1B.* The deletion of case 3 also includes *SIRT5*, another epigenetic regulation gene, and case 2 and 1 also include *ATXN1*. The impact of these genes on the *JARID2* DNA methylation episignature was previously unknown. However, our results demonstrate that all four cases clustered with the already established JARID2-neurovelopmental disorder episignature. 

However, one individual with a deletion spanning exon 6–18 (case 8 from Verberne et al.), initially suspected to have a pathogenic variant, did not exhibit the methylation episignature. The reason for this discrepancy remains unclear. One possible explanation is that this particular deletion has a distinct effect on DNA methylation across the genome, causing it not to align with cases that have more similar functional consequences. A less likely case is that it could also suggest that the *JARID2* episignature lacks complete penetrance in all cases [1]. Further research is necessary to shed light on this unexpected finding. Additionally, we confirmed the same negative results for three previously assessed VUS cases, using the expanded episignature [6]. 

The CNV cohort consisted of four cases with large CNVs involving multiple genes in addition to *JARID2*. All four participants were diagnosed with *JARID2*-neurodevelopmental syndrome based on their phenotype and the presence of deletion of *JARID2*. Case 3 carried a deletion of only exon 1 of *JARID2*. However, there are multiple transcripts of *JARID2* known that include alternative transcriptional start sites (Supplemental Figure S4). Although the largest alternative transcript is not covered by the deleted region detected in case 3, it is possible that the deletion disrupts the gene promoter and impacts *JARID2* transcription in cis. An alternative explanation is that exon 1 of *JARID2* is functionally essential, and therefore the primary cause of the associated episignature and the phenotype, which warrants further investigation. Earlier research showed that deletions of the start site and first exons of haploinsufficient genes are known to be pathogenic in many instances if there are no alternative start sites. The so-called start-loss variants can directly affect the start codon, and their effect on the final protein structure has an influence on the phenotype of patients [12]. This effect is assumed to be similar to patients that have whole-gene deletions of *JARID2* [6]. Taken together, with the similarity in phenotype of case 3 in comparison with the others and the positive signature, this may indicate that *JARID2* is a critical gene within the 6p22–p24 microdeletion region, and that the aberrant methylation is driven by genetic variations involving *JARID2* [1,2,4,5,13]. 

During this analysis, we defined a larger subset of DMPs (n = 628) as representing the global DNA methylation changes in affected *JARID2* cases. Within this subset, we identified two hypomethylated regions, both of which were located in promotor regions and CpG islands. Notably, one of the DMRs overlapped a region containing HOX genes (*HOXA-AS3*, *HOXA3*, *HOXA5*, *HOXA6*). HOX genes are recognized for their significance in embryonic bone, tissue, and organs, and they have been implicated in seizure syndromes [14], mirroring the involvement of *HOX* genes in *JARID2* syndrome. In order to evaluate any effect of the detected DMRs on gene expression, we queried the iMETHYL webtool (http://imethyl.iwate-megabank.org) (accessed on 14 September 2023). Here, we concluded that the DMR annotated to chr7 may be negatively associated with expression of the *HOXA5* gene, and that the DMR annotated to chr17 indicated only a very low negative association with MIR4740 gene expression. Furthermore, *JARID2* DMPs also exhibited overlap with the PCR2 complex episignature that encompasses Cohen–Gibson syndrome (COGIS) and Weaver syndrome (WVS) DMPs. Both COGIS and WVS result from pathogenic variants in the *EED* and *EZH2* genes and are also known to be associated with seizures [15,16]. 

*JARID2* patients demonstrated the highest overlap in DMPs with three cohorts: (1) CHARGE, (2) BAFopathy, and (3) COGIS and WVS. CHARGE syndrome exhibited a ~7% overlap with the *JARID2* cohort, and it is caused by variants in *CHD7*, characterized by multiple congenital anomalies [17]. Notably, the *HOXA5* DMR found in the *JARID2*-neurodevelopmental disorder is also hypomethylated in CHARGE syndrome, which may explain some of the clinical overlap observed between *JARID2*-neurodevleopmental disorder and CHARGE [14]. BAFopathy presented with a ~4% overlap, including *ARID1A*, *ARID1B*, *SMARCB1*, *SMARCA2*, and *SMARCA4*, and includes several neurodevelopmental disorders caused by variants in genes within the BRG1/BRM-associated factor (BAF) complex [18]. The PCR2 complex episignature, which includes Cohen–Gibson syndrome (*EED)* and Weaver syndrome (*EZH2*), presented with a ~4% overlap in DMPs. *JARID2* plays a crucial role during the recruitment and activation of the PRC2 [4,5,11]. The phenotypes of PRC2 and *JARID2*-neurodevelopmental disorders partially overlap; for example, both syndromes may involve ID, seizures, and developmental delay including speech delay [15]. When comparing only the top 500 DMPs detected in the *JARID2* episignature with the previously mapped EpiSign^TM^ disorders, the *JARID2* DNA methylation episignature showed the highest similarity with the CSS9 (*SOX11*) episignature. Proteins encoded by *SOX11* and *JARID2* play crucial roles in multiple developmental processes and belong to the same family of transcription factors, leading to changes in gene expression. Variants in *SOX11* can cause ID, microcephaly, ocular malformation, hypogonadotropic hypogonadism, and dysmorphic features [19,20]. However, it is important to note that in this study, we have not presented supporting evidence of direct regulation by *JARID2* of each of the classifier DMPs, associated DMRs, or their annotated genes.

## 4. Materials and Methods

### 4.1. Subjects and Study Cohort

In addition to the cohort described previously by Verberne et al. [6] (Supplementary Table S1), this study includes four cases with multi-gene CNVs including *JARID2* and one individual with an SNV variant (NM_004973.4: c.1400_1425del, p. (Ala467Glyfs*48)). All cases were identified in a clinical diagnostic setting through microarray analysis or whole-exome sequencing (WES). Variants were classified as pathogenic following the guidelines of the American College of Medical Genetics [21,22].

Episignature discovery included all cases, except for the variant of uncertain significance (VUS) that was used in later episignature validation assessments. 

### 4.2. Sample Processing

DNA from peripheral blood was isolated according to standard techniques. DNA methylation analyses were performed with the Illumina Infinium methylation EPIC bead chip arrays (San Diego, CA, USA) according to the manufacturer’s protocol. Data analysis was performed at the Verspeeten Clinical Genome Centre at London Health Sciences Centre, Canada. Analysis and discovery of the DNA methylation episignature were based on the laboratory’s previously published protocols [10,11]. To summarize, intensity data files (idat) generated after the EPIC array containing methylated and unmethylated signal identities were imported into R (version 4.2.3) and normalized using background correction with the R package minfi (version 1.44.0) [23]. Prior to analyses, the following probes were removed from the dataset: probes with detection *p* value > 0.1, probes located on chromosomes X and Y, probes with single-nucleotide polymorphisms (SNPs) at or near the CpG interrogation site, or single-nucleotide extension sites and probes known to cross react with other genomic regions. After the latter data-cleaning procedure, 772,557 probes remained for data analyses. Samples that contained more than 5% failed probes (*p*-value > 0.1, calculated by the minfi package) were excluded. Next, principal component analysis (PCA) was used to investigate batch structure and to detect case or control outliers. Controls were randomly selected from the EKD [24], though matched by age, sex, and array type using the Matchlt package (version 4.5.2) [25] at a ratio of 1:5. Using the limma package (version 3.54.2) [26], methylation levels for each probe (beta values) were transformed to M-values by logit transformation and linear regression applied to identify differentially methylated probes (DMPs). Finally, estimated blood cell proportions were integrated as confounding variables into the model matrix [27]. As described in the minfi package, the following blood cell types were used as covariates: CD4+, CD8+, natural killer, monocytes, granulocytes, and B-cells. *p*-values were moderated using the eBayes function in the limma package.

### 4.3. Probe Selection and Episignature Classifier Construction

The probe selection and episignature classifier construction method is described previously by Levy et al. [11]. To summarize, probe selection parameters were optimized on the cohort size and signal differences to improve the separation between controls and cases using hierarchical clustering and multidimensional scaling (MDS) plots. Parameters used were: a probe score, the area under the receiver’s operating curve (AUC), and a probe-to-probe methylation correlation. First, a probe score was created with the help of multiplying the absolute value of the mean methylation difference by the negative value of the log-transformed Benjamini–Hochberg-adjusted p value. The probes that received the highest scores were selected, and receiver-operating characteristic (ROC) curve analysis was implemented. Next, the Pearson’s correlation coefficients for the selected probes were calculated, and we removed highly correlated probes. Then, we used the final set of selected probes to perform hierarchical clustering with the R package ggplot2 (version 3.1.3). MDS was performed by scaling of the pairwise Euclidean distance between samples. To calculate the robustness of the episignature, we performed twelve rounds of leave-one-out cross-validation. In each round, one *JARID2* sample was used for testing, and the remaining samples were used for probe selection. Finally, the R package e1071 (version 1.7-13) was used to train a support vector machine (SVM) classifier and to construct a multiclass prediction model. The SVM was trained against all control samples in the EKD. We used 75% of control samples for training and the other 25% for testing, yielding a prediction score termed the methylation variant pathogenicity (MVP) score. The latter was repeated four times, and an average MVP score was obtained for each sample. This methylation variant pathogenicity (MVP) score predicts the probability that the methylation pattern of a sample matches with the given episignature. Scores closest to one indicate the highest probability. 

### 4.4. Annotation of the Global JARID2 DNA Methylation Profile and Correlation to the 56 Neurodevelopmental Disorder Episignatures on EpiSign™

The annotation of the global *JARID2* DNA methylation profile and correlation to the 56 EpiSign™ v3 classifier disorders were based on our previously published methods [10]. To summarize, we produced heatmaps and circos plots to determine the overlapping percentage of DMPs between the *JARID2* episignature and the 56 other neurodevelopmental conditions on the EpiSign^TM^ clinical classifier. All DMPs were used in calculating the overlap percentage. Heatmaps were plotted with the R package pheatmap (version 1.0.12), and circos plots with the R package circlize (version 0.4.15) [28]. To find the genomic location of the DMPs, probes were defined in relation to CpG islands (CGIs), and genes with the R package annotate (version 1.76.0) [29], AnnotationHub (version 3.6.0), and hg19_genes_intronexonboundaries. CGI annotations covered CGI shores from 0–2 kb on both side of CGIs, CGI shelves from 2–4 kb on both side of CGIs, and inter-CGI regions encompassing all remaining regions. For gene annotations, promoters included the region up to 1 kb upstream of the transcription start site (TSS), and promoter+ included the region 1–5 kb upstream of the TSS. Annotations to untranslated regions (5′-UTR and 3′-UTR), exons, introns, and exon/intron boundaries were merged into the “gene body” category. P-values were obtained for both annotation categories, genes and CpG islands. We performed clustering analysis on the combined top N DMPs for all the cohorts described earlier by Levy et al. [10] to find relationships between all the cohorts without prejudice due the number of selected DMPs. This rated the top 500 DMPs for each cohort, ranked by *p*-value. When cohorts had less than 500 DMPs, all the DMPs were used. Finally, the similarities and distance between the cohorts were visualized on a tree-and-leaf plot, which was generated with the R package TreeAndLeaf (version 1.10.0). This plot showed additional information that includes the global mean methylation difference and the total number of DMPs identified in each cohort. 

### 4.5. Differentially Methylated Regions

To find DMRs, we used the R package DMRcate (version 2.12.0) [30]. We only considered regions that contained at least five adjacent significantly different CpGs within 1 kb, with a minimum mean methylation difference of 5% and a Fisher’s multiple comparison *p*-value < 0.01. 

## 5. Conclusions

In this study, we demonstrated that large multi-gene CNVs including *JARID2* exhibit the same DNA methylation episignature as intragenic variants of *JARID2*. This provides evidence supporting *JARID2* as the primary gene responsible for the aberrant DNA pattern in microdeletions of the 6p22–p24 region. We also refined the genomic coordinates for the *JARID2* episignature in 6p22–p24 deletions. Furthermore, we conducted comparative functional analyses with 56 other neurodevelopmental conditions, indicating potential interconnections with *JARID2*. Importantly, the *JARID2* episignature can be employed not only for the diagnosis and reclassification of VUS in intragenic *JARID2* variants but also for microdeletions involving *JARID2* in the 6p22–p24 region.

## Figures and Tables

**Table 1:** Molecular and clinical details of our CNV cohort.

Patient #	1	2	3	4	Summary of This Report	Verberne et al. (2022) [6] (n = 8)
**Variant information**						
Variant type	Deletion	Deletion	Deletion	Deletion		
Variant	6p22.3 (14571015_16248244)x1	6p22.3 (14571015_16381865)x1	6p22.3–24.2 (11327614_15291611)x1	6p22.3–24.2 (9796651_19501625)x1		
Platform	WES and confirmed by Array-CGH	Array-CGH	Array-CGH	Array-CGH		
Inheritance	Not present in their siblings, parents deceased	dn	dn	dn		
Classification	LP	P	P	P		
**General information**						
Gender	M	M	M	M		
Age (years)	28	24	7	10		
**Clinical information**						
***Development/behavior***						
Intellectual disability	+	+	+	+	4/4 (100%)	6/7 (85.5%)
Developmental delay	+	+	+	+	4/4 (100%)	8/8 (100%)
Behavior abnormalities	+	+	+	+	4/4 (100%)	3/8 (37.5%)
Autistic features	+	+	+	+	4/4 (100%)	4/8 (50%)
ASD diagnosis	No formal ASD diagnosis	No formal ASD diagnosis, autistic traits	No formal ASD diagnosis	+	2/4 (50%)	2/8 (25%)
***Neurologic***						
Hypotonia	+	−	+	−	2/4 (50%)	2/8 (25%)
Gait disturbance	−	−	−	−	0/4 (0%)	2/8 (25%)
MRI abnormalities	−	−	−	+	1/4 (25%)	3/3 (100%)
***Dysmorphism***						
Broad forehead	+	−	−	+	2/4 (50%)	3/8 (37.5%)
High anterior hair line	−	+	−	−	1/4 (25%)	5/8 (62.5%)
Prominent supraorbital ridges	+	−	−	−	1/4 (25%)	1/8 (12.5%)
Deep set eyes	+	−	−	−	1/4 (25%)	4/8 (50%)
Infraorbital dark circles	+	+	−	−	2/4 (50%)	4/8 (50%)
Midface hypoplasia	+	-	+	−	2/4 (50%)	1/8 (12.5%)
Depressed nasal bridge	−	-	−	+	1/4 (25%)	2/8 (25%)
Bulbous nasal tip	−	+	+	+	3/4 (75%)	3/8 (37.5%)
Short philtrum	+	−	−	−	1/4 (25%)	3/8 (37.5%)
Full lips	+	−	+	+	3/4 (75%)	2/8 (25%)

Note: M—male, dn—de novo, ASD—autism spectrum disorder, CGH—comparative genomic hybridization.

## Data Availability

The raw DNA methylation data are available on reasonable request.

## Supplementary Materials

The following supporting information can be downloaded at: https://www.mdpi.com/article/10.3390/ijms241814240/s1.
