# Supplementary material for: Functional Insight into and Refinement of the Genomic Boundaries of the JARID2-Neurodevelopmental Disorder Episignature
Source: Int J Mol Sci. 2023 Sep 18;24(18):14240. doi: 10.3390/ijms241814240 (PMC10531903; doi:10.3390/ijms241814240)
Supplement: Supplementary file 1 [file ijms-24-14240-s001.zip › Supplementary figures.pdf]

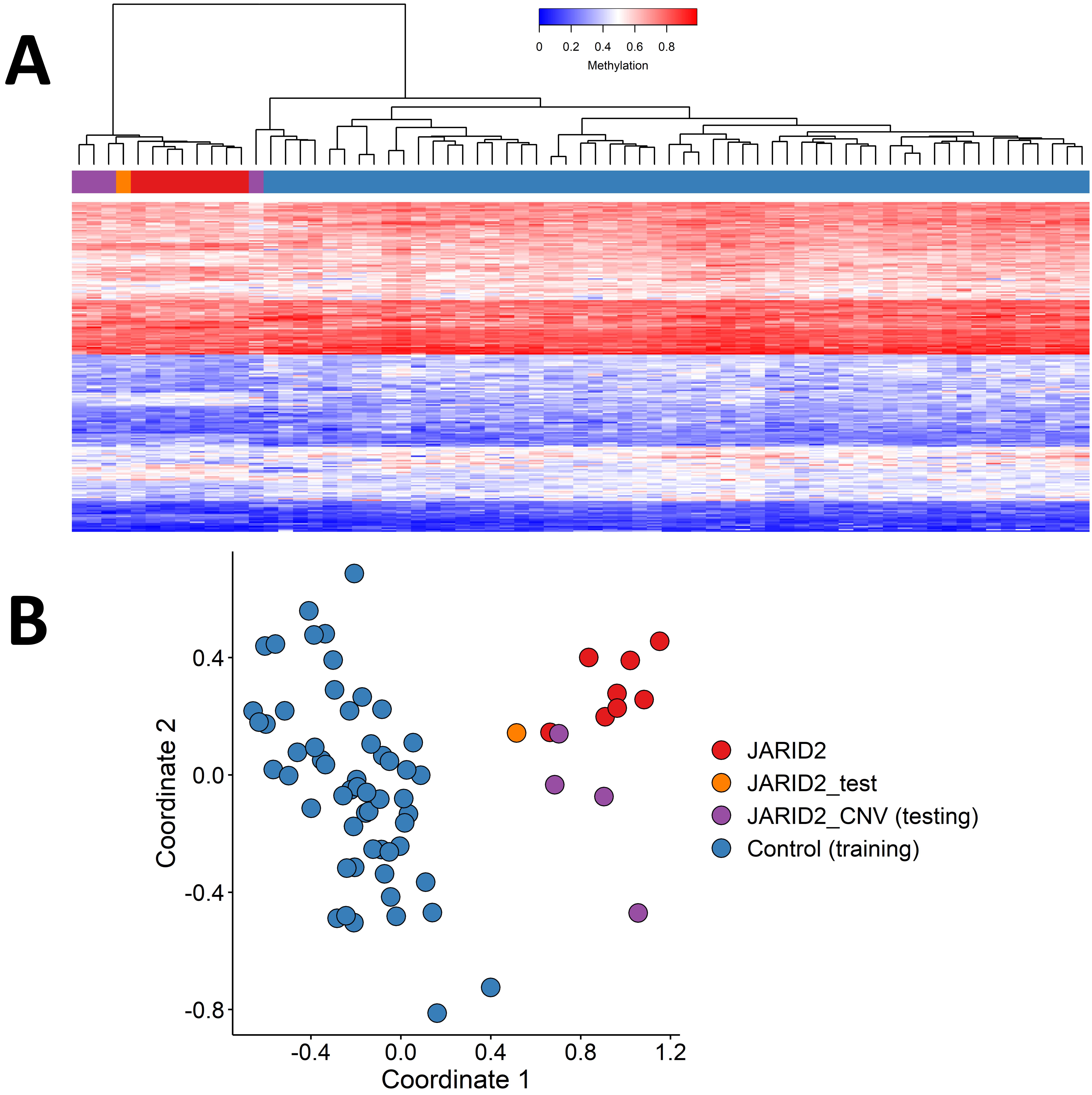

Supplementary Figure S1 ; Validation of the earlier discovered JARID2-neurodevelopmental disorder episignature. A) Euclidean clustering heatmap of the cases in red and the controls in blue. Rows of the heatmap correspond to the selected probes for the identification of the episignature and the columns represent the cases and controls. The methylation levels are colored to show the intensity values, with 0 as blue and 1 as red. We added the CNV patients in purple and the SNV validation case in orange as testing samples. **B)** Two-dimensional multidimensional scaling plot. The x and y axis represent the first and second dimension of the output (Coordinate 1 and 2 respectively).

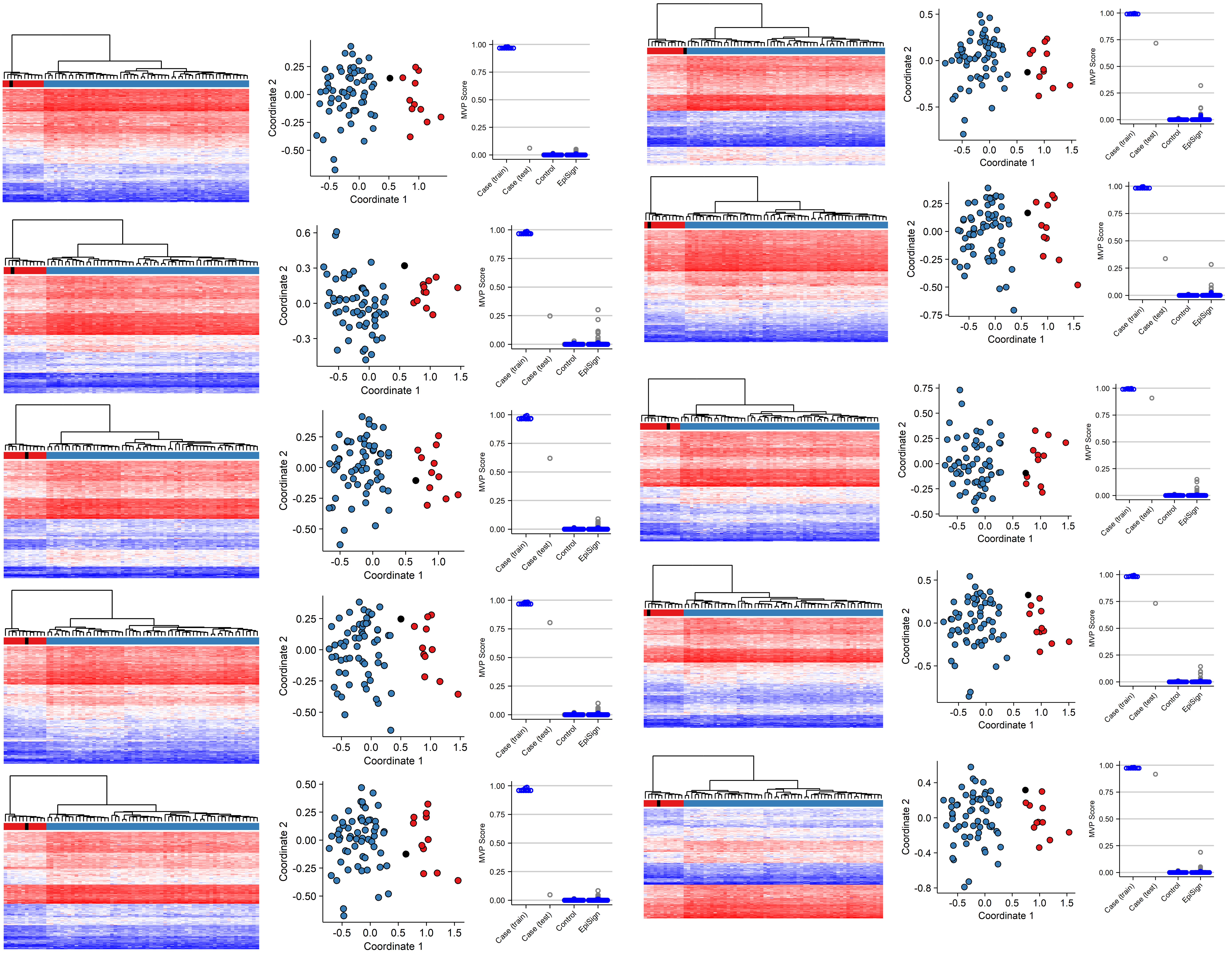

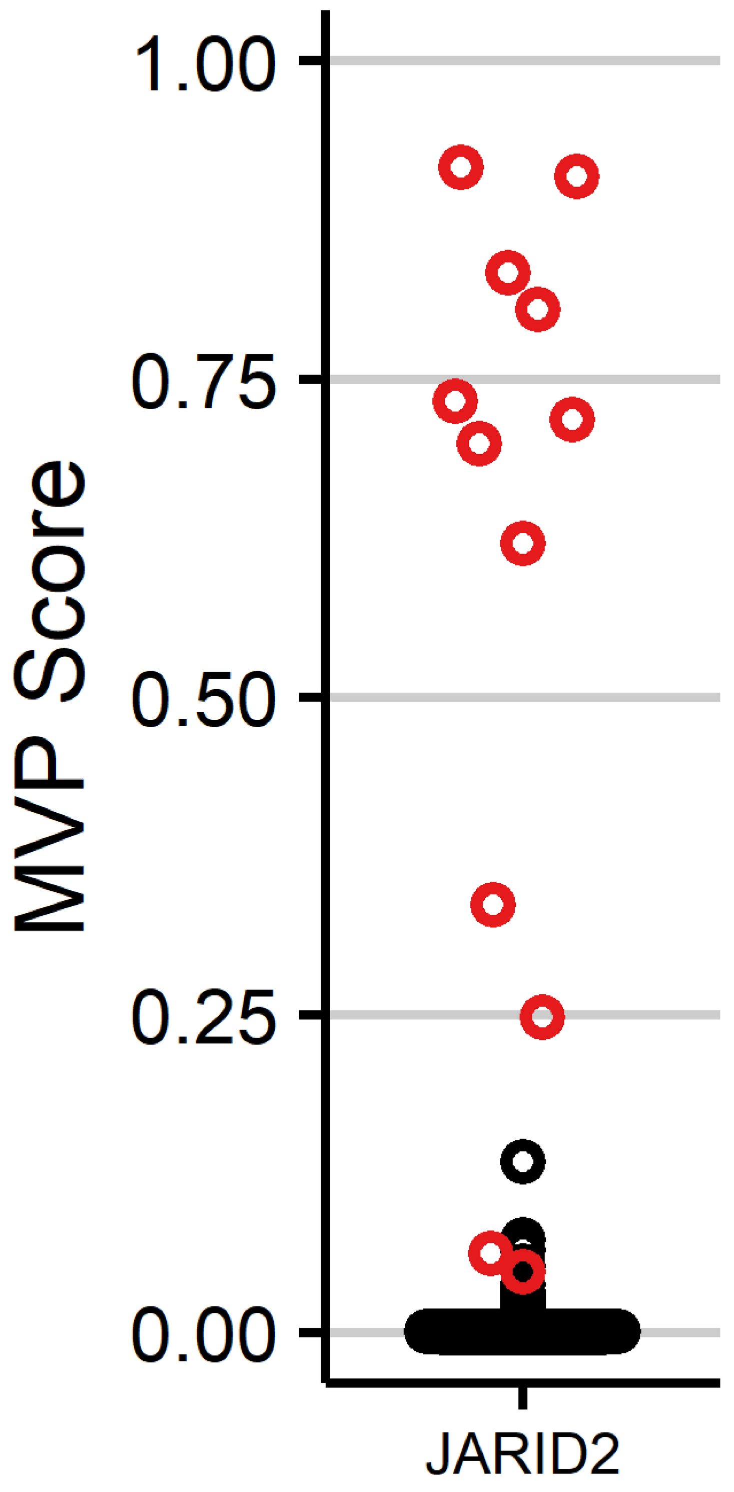

Supplementary Figure S2 ; Leave-one-out cross validation. In each cross-validation set, a singe test case sample (dark blue) is used as testing. The other *JARID2* cases used for episignature training are shown in red and control training sample shown in blue in the heatmap and MDS plots. The last plots demonstrate the MVP scores of the Support Vector Machine (SVM) classifier model that was trained using the selected *JARID2* episignature probes from training cases, 75% of controls and other EpiSign samples (blue). The remaining 25% of controls and other disorder samples were used as testing alongside the *JARID2* case (grey).

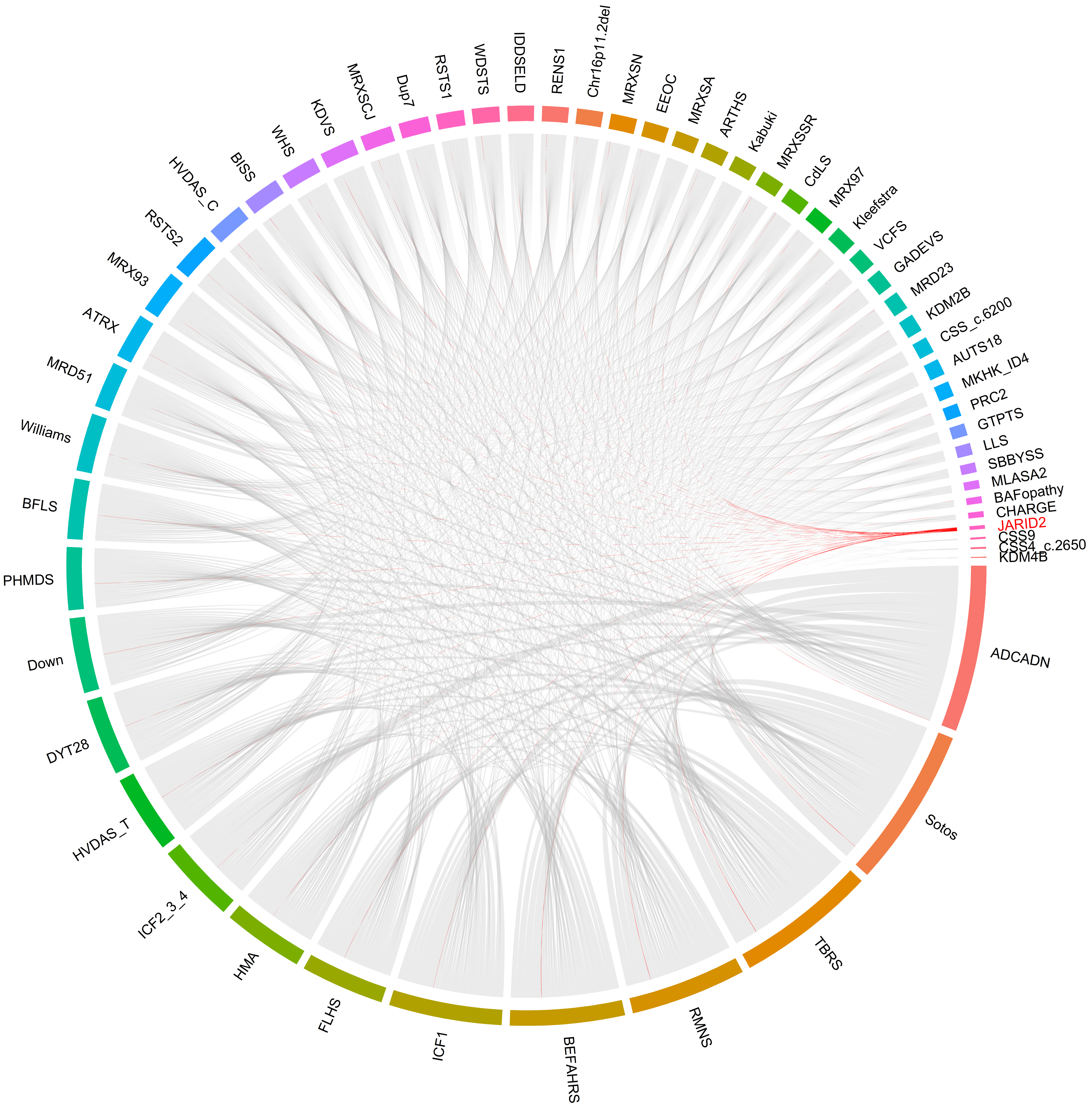

Supplementary Figure S3. Circos plot representing the probes shared between each pair of cohorts. The thickness of the connecting lines indicates the number of probes shared between the two cohorts.

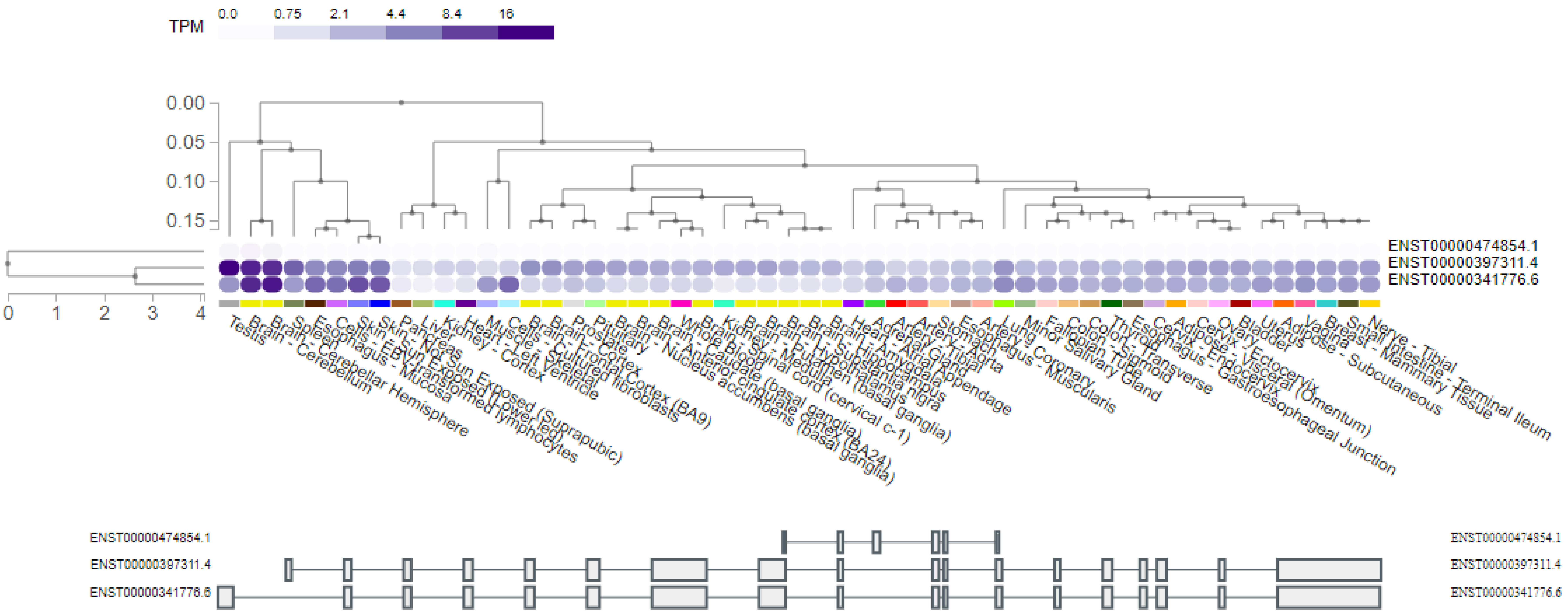

Supplementary Figure S4; Isoform Expression of JARID2: ENSG00000008083.13 jumonji and AT-rich interaction domain containing 2 [Source:HGNC Symbol;Acc:HGNC:6196]. The Genotype-Tissue Expression (GTEx) Project was supported by the Common Fund of the Office of the Director of the National Institutes of Health, and by NCI, NHGRI, NHLBI, NIDA, NIMH, and NINDS. The data used for the analyses described in this manuscript were obtained from: the GTEx Portal on 09/01/2023 and dbGaP accession number phs000424.vN.pN on 09/01/2023.
